# 

**Published:** 1888-12-15

**Authors:** 


### THE STUDENT'S COLUMN.

### Materia Medica.*

When a book of this kind attains to the age and dignity of a fifth edition, the reviewer, even although writing with a bradawl dipped in vitriol, cannot truthfully say much in disparagement of the volume. For there are so many books of a similar import that it is certain no particular one would succeed without special merits. For instance, we have that venerable (as regards parentage at least), mysterious, and very incomplete work, the "British Pharmacopœia," a work which has, however, been rendered complete by "Squire's Companion" volume. The foolish idea has occurred to us that by amalgamating these two works the double publication would be avoided and a complete work obtained, while the present tax on medical practitioners of being obliged to purchase two would be removed. "Squire's Companion" certainly supplies a want which the "British Pharmacopœia" does not supply. For, in addition to information how to make drugs, Squire gives the medical properties, and for what maladies or purposes medicines are chiefly used. Not one medical practitioner in a hundred manufactures his own drugs; he obtains them from chemists, and the "British Pharmacopœia" is of little use to him. No doubt a committee of eight, chosen from that august assembly the "Council of Medical Education and Registration," especially when aided by three editors, could have produced a better book than the "British Pharmacopœia." Why that portentous volume should not give all the information to be found in "Squire's Companion," or even in "Squire's Small Companion," or in Waring's "Manual of Therapeutics," or in the book before us, "Craig's Manual of Materia Medica"—why the "British Pharmacopœia" should not give such additional information there is no valid reason. If it is stated that it is not the intent and purport of the "British Pharmacopœia" to afford such information, then we say the intent and purport of the work ought to be altered, and Squire and Craig are the men to do it.

The primary object of Dr. Craig's manual is to assist students attending the author's classes of Materia Medica and Therapeutics, and of Pharmacy. The fifth edition has been carefully revised and in several parts rewritten, and there is no doubt that it will be found useful, not only to the members of the author's classes, but wherever the subjects are taught. In order to show the plan of the work we take a preparation in ordinary use, viz., Potassii Iodidum, or K. I., which, it is stated, is obtained by acting on solution of potash with iodine. Next, the characteristics of the salt and the tests of purity are given; then the dose, and the adulterations and the means of detecting them. The preparations into which iodide of potassium enters are next mentioned, and the whole is followed by an article on the action and uses. To this part of the treatment of the subject much care has evidently been given, and the author not only records the generally-received opinions of the action and uses of the various drugs, but also in many instances adds his own experience.

Part 2 of the Manual consists of "Medicines Arranged According to their Actions," and part 3 treats of "Particular Forms of Medicines," such as cataplasms, decoctions, infusions, lotions, mixtures, etc. Part 4 is a still more valuable addition, and consists of "Posological Tables," being a tabular arrangement of all the medicines contained in the "British Pharmacopœia," showing doses, action, and method of administration. This information is conveyed in columns headed "Medicine—Dose for Adult—Action and Uses—Form of Administration." At random we take the first medicines on pp. 404, 405, which are Acidum Hydrocyanicum dilutum, and Aloin. From the tables we learn that the dose of the former is from two to eight minims, that it is a poison, and sedative in cough, and used in a draught with mucilage or syrup. The dose of aloin is from half to two grains—cathartic—given in a pill. Next, there is a tabular statement, showing the symptoms and treatment of poisoning. Following this there is an "Index of Diseases," with their most important remedies. The last part is an essay on prescription writing, with an appendix of contracted terms in common use. *En passant* we may observe that we really wonder abbreviated Latin is not dropped, for it puzzles most people, especially when the writing is bad, as much as an Etruscan inscription. Sulph. mag. and sol. sen. are not more effectual than they would be if designated by English terms. We presume it is a remnant of that *omne ignotum pro magnifico* which the profession once affected. In conclusion, enough has been said to show that Dr. Craig's work is a veritable *multum in parvo*, and useful not only to students, but to practitioners also.

* 'A Manual of Materia Medica and Therapeutics," by William Craig, M.D., C.M. Ed., F.R.S.E., F.R.C.S. Ed., Lecturer on Materia Medica, Edinburgh School of Medicine. Fifth edition, revised and enlarged. (E. and S. Livingstone, 15, Teviot Place, Edinburgh.)

**Notice to Advertisers and Subscribers.**

All Advertisements, Orders for Copies of The Hospital, and Business Communications should be addressed to The Publisher, *not to the Ed tor*, The Hospital, 38, Old Jewry, E.C.

**To Residents, Secretaries, and Matrons.**

The Editor will be glad to have the Regulations and each Report as issued by Asylums, Hospitals, Convalescent and Nursing Institutions, as well as those of other Charities, and an early copy in duplicate of all Appeals and Festival Notices, etc., addressed to *The Editor, The Lodge, Porchester Square, London, W.* Any superintendent, secretary, matron, or other chief official who regularly sends such information may be registered, on application to the Publisher, to receive free of cost a cover for binding The Hospital in half-yearly volumes.

### Scraps and Gleanings.

Hospital Saturday collection at Horncastle amounted to £16.

A Ladies' Committee has been appointed at the Victoria Hospital, Burnley.

The fatal cases of measles are increasing in London. They have now reached 140 a week.

Driffield Cottage Hospital treated 43 in-patients and 41 out-patients in the year.

The Frenchman named Jacques has completed in safety his fast of thirty days at Edinburgh.

It is proposed to close Thoresby Street, Leeds, to facilitate the extension of the infirmary.

The matron of Nottingham General Hospital appeals for articles for the patients' Christmas-tree.

The captain and officers of the s.s. *Roslin Castle* have contributed £90 to the Hartlepool Hospital.

The secretary of the Jews' Hospital and Orphan Asylum is the latest antagonist to the voting system.

In London and the suburbs, Nov. 25th was kept as Temperance Sunday at Nonconformist places of worship.

Dr. Henry Miller, of Wilmington, America, has made three millions of dollars by the sale of patent pills.

Professor Garcia gave a concert last week in aid of Luton Cottage Hospital. The audience was small.

The annual concert in aid of Caterham Cottage Hospital was given on November 26th by the Rev. F. J. Roe.

A movement has been initiated at Woolwich for the erection of a suitable memorial to the late Colonel Duncan.

Cumberland Infirmary has received a legacy of £500 under the will of the late Mr. W. Skelton, of Forest Hill.

The Ballymena Dispensary District is to be divided, and two medical men appointed in place of Dr. Ross, resigned.

It is averred that Brooklyn Asylum is overcrowded, and that some of the patients are hidden away in noisome cellars.

Dr. Shettle and Dr. Kule has been elected respectively surgeon and assistant surgeon of the South Hants Infirmary.

Mr. Gladstone has given it as his opinion that under the present Vaccination Act there is both hardship and inequality.

Mr. Owen Lankester lectured at Newington last week, for the National Health Society, on the care of children's health.

Unpleasant rumours being current concerning the Borough Hospital, Stockport, the committee have asked the matron to resign.

Dr. Buchan, of Plymouth, has consented to act as treasurer of a Samaritan Fund in connexion with the South Devon Hospital.

Dr. Hussey has resigned the post of surgeon to the Brighton Borough Hospital, and Dr. Newsholme has been appointed in his place.

The Consumption Hospital, Ventnor, is supposed to keep away ordinary invalid visitors who might otherwise reside in that town.

A Bohemian smoking concert in aid of King's College Hospital, was lately given at the Portman Room. There were several ladies present.

A bazaar will be held at the Town Hall, Chelsea, on December 11th and three following days, in aid of the Jubilee Hospital, Richmond Road.

Dr. Mannix has been elected medical officer of Cahirciveen. It was hoped two medical men would have been appointed, as the district is large.

A concert was given at the Mechanics' Institute, Jarrow, on November 28th, in aid of the Liddell Provident Dispensary. The Mayor was in the chair.

Over 450 persons attended the Fancy Dress Ball at Brighton, in aid of the County Hospital. Mrs. Edmund Vallance was honorary secretary.

The sanitary condition of Manchester, and its abnormally high death rate (2·5 per cent., while that of London is 0·9) is being discussed in the local papers.

A correspondent writes to the "Hampshire Chronicle" to point out that the Victoria Hospital is becoming a much more expensive institution than was expected.

The Prince of Wales has forwarded a large quantity of game for the use of the patients of St. George's Hospital, London, and to the Great Yarmouth Hospital.

Only 49 out of 125 congregations responded to the appeal of the Belfast Hospital Sunday Fund. This looks as though there was something wrong at the hospital.

The Rev. J. T. Dove gives facts about the outbreak of typhoid fever at the Lincolnshire Asylum. One hundred patients, nurses, and doctors were attacked, and 19 died.

The attendance at the annual meeting of the subscribers to Guest Hospital, Dudley, was very small. The report was satisfactory, there being a balance on the right side.

Estimates are about to be selected for alterations on Campie House, Musselburgh, which is to be used as a convalescent home in connection with the Edinburgh Fever Hospital.

Mr. Quarrier's Orphan Homes Bridge of Weir, have lately been largely improved. A hospital and store-room have been added, and a peal of bells, which cost £7,000, have been fitted to the church.

We regret to see the vicar of St. Andrew's, Gorleston, refuses to hand over to the Cottage Hospital the collection made in his church on Hospital Sunday, unless he gets back an equivalent in out-patients' tickets.

Dr. Collingridge, Medical Officer of Health for the Port of London, has obtained at the Greenwich Police Court, an order for the destruction of nearly one hundred tons of herrings, the cargo of a vessel in the Commercial Docks.

The Society for the Relief of Distress appeal for funds to enable them to make the usual grants to their almoners at this season. They acknowledge the receipt from the Duke of Westminster of £100, and from the Duke of Bedford of £50, and other smaller sums.

### Amusements and Relaxation.

### NEW PUZZLE PRIZE SCHEME.

COMMENCED OCTOBER 6TH, ENDS DECEMBER 29TH, 1888.

On October the 6th a New Prize Scheme was commenced in these columns.

Prizes will be awarded for the best Original Puzzles in Prose or Verse, relating to any of the *Social, Philanthropic, or Political Topics of the day*, and taking the form of Double Acrostics, Anagrams, Square Words, Decapitations, Transpositions, or any other Puzzles which the ingenuity of the competitors may suggest. (*Answers must accompany contributions.*)

*Readers* are requested to send in weekly the Number of Marks they consider each composition to be entitled to, three being the highest score for any one contribution.

Eight Prizes of Standard Books will be given, the choice of the book to be left to the winner.

First, Second, Third, Fourth, and Fifth Prizes for best Original Puzzles of £1 1., 15s., 10. 6d., 7s. 6d., and 5s. each.

First, Second, and Third Prize for Solutions of 15s., 10. 6d., and 5s. each.

N.B.—All contributions must reach the office, 38, Old Jewry, E.C., not later than the *First Post* on *Thursday* in each week.

### PUZZLES FOR SOLUTION.

### Ninth Week.

No. 23. Double Acrostic.

Amusement, Science, and Gossip, all three in me are blended;
But when you come to the first of these three, alas! I'm nearly ended.
A helper to an ancient king.
Here bygone wrestlers had a fling.
A foe to flowers and all things green.
Name of a mighty work I ween.
Camoëns, Spanish heroine this.
An isle the Southern waves do kiss.
Final oft used when heads are bowed.
Beloved Princess with art endowed.

Thanet.

No. 24. Conundrum.

Why is the "Puzzle Page" in The Hospital like a deep thinker?

Patience.

No. 24. Hour Glass Puzzle.

The sands of eighteen eighty-eight
Are quickly following others' fate.
The last few grains have yet to go;
I yet may bring both ice and snow.

. . . . . . . .
. . . . . .
. . . .
. .
. . . .
. . . . . .
. . . . . . . .

1. In me the sun's bright rays are rare.
2. To see that everything is fair.
3. In rivers I'm found everywhere.
4. A measure that makes all things square.
5. Spend here the time that you can spare.
6. A free outlet to the open air.
7. A time when trees of leaves are bare.

Tinie.

### Marks.

| Names. | No. of Puzzles sent in Dec. 6. | No. of Marks Dec. 6. | Total of Marks. | Names. | No. of Puzzles sent in Dec. 6. | No. of Marks Dec. 6. | Total of Marks. |
|---|---|---|---|---|---|---|---|
| Patience | 3 | 4 | 46 | Hercules | — | — | 11 |
| Lady Clara | 3 | 5 | 56 | Gretta | 3 | 4 | 39 |
| Tinie | 3 | 9 | 51 | Dandy Dick | — | — | 5 |
| Bijou | 2 | 6 | 27 | Boadicea | — | — | 7 |
| G. D. | — | — | 15 | Nipper | 3 | 4 | 38 |
| Thanet | 1 | 2 | 30 | Morico | 3 | 7 | 40 |
| Daisy | — | — | 16 | A. M. E. | — | — | 3 |
| Salford | — | — | 6 | John Gilpin | — | — | 4 |

### Answers to Seventh Week Puzzles.

No. 18. Charade. Surge-on=Surgeon.

No. 19. Diagonal Puzzle.

E v y e n t I
i N q u i R y
e a G l E t s
e x p L o i t
b r A v A d o
a N c i e N t
D i a m o n D

No. 20. In-val'id / In'va-lid } Invalid.

### Marks for Solution of Puzzles.

Total Marks.—Ruffles (2), 11; Daisy, 7; Lady Clara (1), 10; Reldas, 9; Mater (1), 6; Condorcet, 7; Tinie (2), 11; Gentian (2), 13; Marguerite, 5; Patience (2), 7; Gretta, 4; Amicus (1), 7; Morico, 3; Scrooge (2), 12; Smut, 2; Thanet (2), 15; Jenny Wren (1), 3; Lauriston, 1.

### Conditions.

I. Every paper sent in must be clearly written, and one side only must be used. All competitors must mark at the head of their papers the number of their competition.

II. Each paper must be signed by the author with his or her real name and address. A *nom de plume* may be added if the writer does not desire to be referred to by us by his real name. In the case of all prizewinners, however, the real name and address will be published.

III. All communications relating to prize competitions should be addressed to "The Prize Editor, The Hospital, 38, Old Jewry, London, E.C." Competitors are requested not to include in letters, etc., to the Prize Editor, communications on matters of other business. All letters must be fully prepaid.

IV. The Prize Editor of The Hospital is the sole judge of the competitions, and his decision is in all cases final and without appeal.

V. The Prize Editor reserves the right to publish any paper sent in, whether it receives a prize or not. He does not hold himself responsible for any MSS. sent, nor can he undertake to return rejected competitions.

